# Impact of UV Aging on the Toxicity and Bioavailability of Inductively Coupled Plasma Mass Spectrometry (ICP-MS)-Traceable Core–Shell Polystyrene Nanoplastics in an In Vitro Triculture Small Intestinal Epithelium Model

**DOI:** 10.3390/toxics13110939

**Published:** 2025-10-30

**Authors:** Satwik Majumder, Lila Bazina, Glen DeLoid, Alvaro G. Garcia, Nubia Zuverza-Mena, Jakub Konkol, George Tsilomelekis, Michael Verzi, Hao Zhu, Jason C. White, Philip Demokritou

**Affiliations:** 1 Nanoscience and Advanced Materials Center, Environmental and Occupational Health Sciences Institute (EOHSI), Rutgers Biomedical Health Sciences, Rutgers University, Piscataway, NJ 08854, USA; sm3397@eohsi.rutgers.edu (S.M.); lb948@gsbs.rutgers.edu (L.B.); gd424@eohsi.rutgers.edu (G.D.); 2 Department of Analytical Chemistry, The Connecticut Agricultural Experiment Station, New Haven, CT 06511, USA; alvaro.g.garcia@ct.gov (A.G.G.); nubia.zuverza@ct.gov (N.Z.-M.); jason.white@ct.gov (J.C.W.); 3 Department of Chemical and Biochemical Engineering, Rutgers University, 98 Brett Road, Piscataway, NJ 08854, USA; jak496@soe.rutgers.edu (J.K.); gt241@soe.rutgers.edu (G.T.); 4 Department of Genetics, Rutgers University, Piscataway, NJ 08854, USA; mv347@rutgers.edu; 5 Center for Biomedical Informatics and Genomics, School of Medicine, Tulane University, New Orleans, LA 70112, USA; hzhu10@tulane.edu

**Keywords:** micro-nanoplastics (MNPs), UV-aging, environmentally relevant MNPs, gold-core polystyrene shell NPs (AuPS25 NPs), polystyrene (PS), toxicity, translocation, small intestinal epithelium (SIE)

## Abstract

A major bottleneck in evaluating the environmental health implications of micro-nanoplastics (MNPs) is the inadequacy of analytical techniques for their precise quantification within complex environmental and biological matrices. Additionally, there is a conspicuous paucity of studies addressing environmentally relevant, photo-aged MNPs. In this study, the effects of UV aging on toxicity and bioavailability were investigated utilizing inductively coupled plasma mass spectrometry (ICP-MS)-traceable 25 nm gold-core polystyrene shell nanoplastics (AuPS25 NPs) and a triculture small intestinal epithelium (SIE) model coupled with simulated digestions to mimic physiological bio-transformations post-ingestion. Employing dynamic light scattering (DLS), transmission electron microscopy (TEM), Fourier-transform infrared spectroscopy (FT-IR), and X-ray photoelectron spectroscopy (XPS), the physicochemical and morphological alterations of AuPS25 NPs as a function of UV exposure time were investigated, revealing significant photo-oxidation within 14 days. Toxicological evaluations demonstrated that, contrasting with un-aged AuPS25 NPs, the digesta from UV-aged AuPS25 NPs at oral concentrations of 4 and 40 µg/mL weakened barrier integrity by ~15% and ~18% and heightened cytotoxicity by ~4.3% and ~5.4%, respectively. Although the NP translocation rates were similar for both aged and un-aged PS NPs, the uptake by SIE of aged AuPS25 NPs was significantly higher, reaching 72.2% at 4 µg/mL and 59.2% at 40 µg/mL. In contrast, less than 0.5% of the un-aged PS NPs at both 4 µg/mL and 40 µg/mL were taken up by SIE. These findings highlight the imperative to integrate environmentally aged MNPs into toxicological assessments, as they facilitate “real-world” MNPs. Finally, the use of ICP-MS-traceable core–shell MNPs enables the identification and quantification of PS MNPs in cell lysates and biological media via ICP-MS, showcasing the use of such a tracer MNP approach in cellular uptake and in vivo biokinetic studies.

## 1. Introduction

The massive production and inadequate disposal of plastics have led to increased environmental accumulation. Annual plastic production surged from 2 million metric tons in 1950 to 400 million metric tons in 2022 [1]. The recycling rate for plastics is only 9%, with about 12% being incinerated, while the remainder is deposited directly into the landfill, aquatic, atmospheric, and terrestrial environments [2]. Over time, this discarded plastic waste undergoes mechanical, thermal, and photo-oxidative degradation, resulting in the creation of micron-to-nanometer-scale plastic fragments termed micro-nanoplastics (MNPs) [3]. MNPs are also generated during municipal incineration of plastic trash, as well as processes associated with the wear and tear of plastic products [3,4], including the friction of tires on road surfaces [5], abrasion of synthetic textiles during laundering [6], and natural weathering of plastic-based materials such as paints, coatings, and emissions from toner-based printing and 3D printing technologies [7,8,9]. As a result, MNPs have become ubiquitous contaminants of air, soil, water, and the entire food web [2,10,11].

Recent studies have reported the detection of MNPs in a wide variety of foods [12], including prawns (16 µg/g) [13], packaged chicken (18–164 particles/kg) [14], fruits (1 × 10^5^–2 × 10^5^ particles/g) [15], table salt (50–280 particles/kg) [16], honey (40–160 particles/kg) [17], and bottled water (2500–10,000 items/L) [18], among many others. In addition, the ability of MNPs to be taken up by and translocated to the edible portions of plants was recently confirmed [19]. Consequently, ingestion has emerged as one of the principal routes of human exposure. Given that multiple studies in animal models have shown that MNPs are efficiently absorbed within the gastrointestinal tract (GIT) [20,21,22,23], such ingestion exposures are likely to result in systemic exposure and widespread distribution of ingested MNPs. The ability of MNPs to bypass biological barriers, facilitating their accumulation in various organ systems, has been documented both in animal and human biomonitoring studies [24]. Notably, MNPs consisting of polyethylene (PE), polystyrene (PS), and polyvinyl chloride (PVC), among others, have been found in multiple human organs and tissues, including liver, kidney, heart, spleen, brain, lung, uterus, placenta, testes, ovaries, bone marrow, and blood [22,24,25,26,27,28,29]. Importantly, cellular and animal model exposure studies have demonstrated cytotoxicity, reduced viability, oxidative stress, morphological changes, mitochondrial dysfunction, lysosomal damage, apoptosis, DNA damage, inflammation, metabolic dysfunction, and impaired barrier function in response to MNP exposures [3,30,31,32,33,34,35,36,37]. For instance, we have previously reported translocation and accumulation of 20 nm PS spheres in the placenta and multiple fetal organs, including brain, liver, heart, intestine, and kidney, within 24 h after a single oral gavage exposure in pregnant rats, indicating that ingested nanoscale PS can breach both the intestinal barrier and the maternal–fetal barrier of the placenta [24]. We also previously reported that 25 and 1000 nm ingested PS MNPs impaired barrier function and caused DNA damage in an in vitro model of the small intestinal epithelium (SIE) in a size-, dose-, and exposure-duration-dependent manner [30,36].

A serious limitation of the current MNP research literature is that the vast majority of toxicological studies published to date have been conducted using commercially available primary MNPs, most commonly composed of pristine polymers such as PS, PE, polyethylene terephthalate (PET), and PVC [3,30,31,32,34,35,38]. For meaningful insights into the environmental and health implications of MNPs, it is critical to consider the complex physicochemical alterations of MNPs that occur during environmental weathering/aging processes, including mechanical fragmentation, photo-oxidation, and hydrolysis. The transformations, particularly photo-oxidation resulting from exposure to UV radiation, have been shown to significantly alter the surface chemistry and morphology of MNPs, increasing their roughness and the presence of reactive functional groups [39]. These changes correlate with enhanced toxicity, as evidenced by findings in cellular models wherein exposure to UV-aged MNPs resulted in compromised cell membrane integrity and reduced cell viability [40,41]. In recent years, our laboratory and a few other groups have been developing laboratory-synthesized, property-controlled, environmentally relevant MNPs, specifically designed for risk assessment analysis [42,43,44,45]. These MNPs are generated using lab-based platforms that encompass the full range of environmental degradation processes across the life cycle of plastic materials, including mechanical fragmentation, photo-oxidation, and thermal degradation, and thus closely mimic ‘real-world’ environmental secondary MNPs [4,45,46].

Another challenge in MNP risk assessment studies is the lack of robust analytical methods that possess the necessary sensitivity and selectivity to provide accurate speciated quantification of MNPs, as well as characterization of key physicochemical properties that could determine bioactivity and potential toxicity in complex environmental and biological matrices. Until recently, the vast majority of studies reporting on the identification and quantification of MNPs have utilized spectroscopy–microscopy-based techniques such as Raman microscopy, Fourier-transform infrared (FT-IR) microscopy, and laser direct infrared (LDIR) imaging [47]. Despite their utility, these methods are constrained by lengthy processing times and lower size limits that preclude detection and quantification of nanoscale MNPs, resulting in incomplete quantification of total MNP concentrations. Pyrolysis gas chromatography mass spectrometry (Py-GC/MS) has emerged as a useful method for quantitative analysis of total speciated MNPs. However, because it is a destructive method, it cannot provide critical data on particle sizes and counts. In addition, it may lack the sensitivity necessary to accurately quantify MNPs in biological tissues at physiologically relevant concentrations, particularly in the presence of various matrix interferences, which may limit its applicability in toxicological studies [48,49].

The emerging data confirming the presence of MNPs in complex environmental and biological matrices underscores the urgent need for the development of advanced analytical methodologies for quantification purposes. To that end, researchers are increasingly focusing on the development of environmentally relevant “labeled” MNPs [50]. These labeled MNPs are designed to mimic the physical and chemical characteristics of environmentally relevant MNPs while incorporating identifiable and quantifiable elemental or fluorescent markers that are not typically present in natural environmental or biological matrices [51]. However, concerns regarding leachability and photolability in dynamic natural systems have significantly limited their practical application in field and laboratory studies [52]. Alternatively, embedding of metallic ions or nanoparticles within the polymer matrix has been recently explored [53], but concerns have been raised related to the doping of polymers with toxic metals that may result in the leaching of toxic ions [54], resulting in direct toxicity to the cells or organisms being exposed. Alternatively, MNPs can be designed to incorporate a metallic core within an outer plastic shell, thereby preventing leaching of ions or direct contact of cells or biomolecules under investigation with metallic surfaces. Techniques such as inductively coupled plasma mass spectrometry (ICP-MS) can enable the detection and accurate quantification of these metallic components at very low concentrations, providing a more stable and reliable method for studying the behavior and fate of MNPs in complex environmental matrices and biological systems [53,54,55].

To address the above-identified knowledge gaps, in this study, the effect of photo-aging on the physicochemical and morphological properties, toxicity, and bioavailability of ICP-MS-traceable 25 nm sized gold-core shell polystyrene nanoplastics (AuPS25 NPs) was investigated using orthogonal analytical methods and an advanced cellular model of the SIE coupled with simulated digestions. The physicochemical and morphological properties of AuPS25 NPs were systematically characterized as a function of UV exposure time by dynamic light scattering (DLS), transmission electron microscopy (TEM), FT-IR, and X-ray photoelectron spectroscopy (XPS) to assess the environmental impacts of photo-aging. The application of these tracer PS NPs in toxicological assessment was demonstrated using an in vitro transwell triculture model of the SIE, coupled with simulated digestions to reproduce the physiological bio-transformations (corona formation and agglomeration) of ingested MNPs in the gastrointestinal tract (GIT) [56,57]. The uptake and translocation of the PS NPs and the role of photo-aging were quantified by ICP-MS, providing insights into their bioavailability and potential health implications and showcasing the use of such ICP-MS-traceable MNPs in biokinetic studies.

## 2. Materials and Methods

### 2.1. Study Design

**Figure 1:**
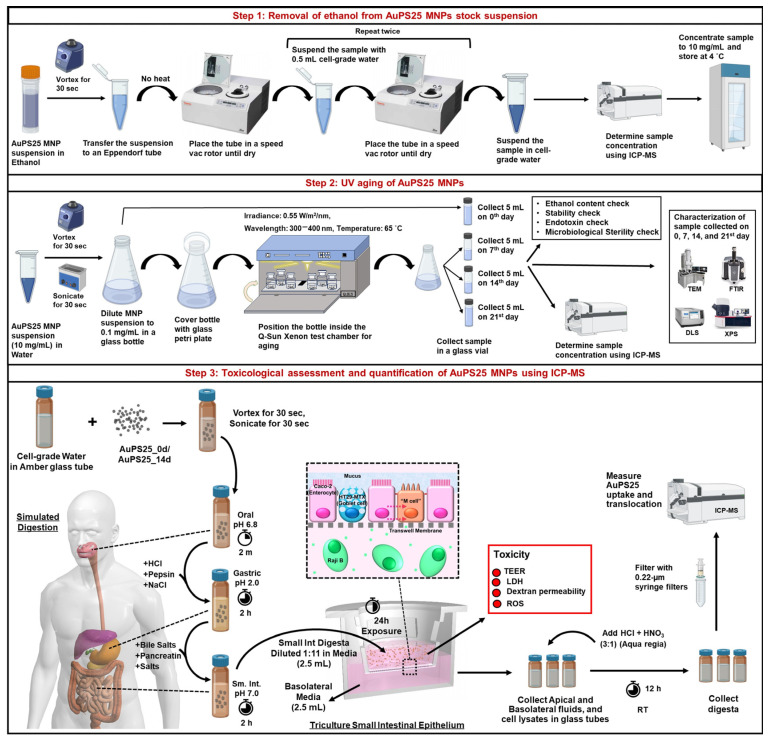
Study design overview.

The study design is illustrated in Figure 1.

Preparation and characterization of NPs: Briefly, 25 nm sized gold core–polystyrene spheres (referred to as AuPS25), suspended in absolute ethanol, were sourced from CD Bioparticles, NY, USA. After evaporating the ethanol utilizing a SpeedVac Vacuum concentrator, the AuPS25 was resuspended in a food model (water, a fasting food model) using water-bath sonication. The AuPS25 NPs were then UV-aged for 21 days using the Q-Sun Xenon UV aging chamber, Westlake, OH, USA. To evaluate the aging effects over time, physicochemical characterization of the samples was performed on day 0 (un-aged) as well as on the 7th, 14th, and 21st days (aged) of UV exposure using DLS, TEM, FT-IR, and XPS. The ethanol content, endotoxin levels, and microbiological sterility were also assessed on the prepared AuPS25 samples used in toxicity testing to avoid potential confounding sources of toxicity beyond the AuPS25 NPs themselves.

Toxicity testing: Un-aged and aged AuPS25 NPs suspended in water were subjected to a three-phase (oral, gastric, and small intestinal) simulated digestion process to replicate the physicochemical transformations that would occur during in vivo ingestion, which can significantly affect biointeractions [30,56,57]. The resulting small intestinal (SI) digesta containing digested NPs was applied to an in vitro triculture SIE model, including cells representing intestinal epithelial enterocytes (matured Caco-2 cells), mucus-secreting goblet cells (HT29-MTX), and microfold or M-cells (Caco-2 cells transformed by Raji B feeder cells). The impact of ingested NPs on the SIE was investigated by toxicological analysis, including oxidative stress (generation of reactive oxygen species (ROS)), lactate dehydrogenase (LDH) release, trans-epithelial electrical resistance (TEER), and dextran permeability. Lastly, the Au cores in AuPS25 were quantified using ICP-MS to provide accurate measurements of AuPS25 uptake by and translocated through the triculture SIE.

### 2.2. Removal of Ethanol and Dispersion of AuPS25 NPs in a Food Model (Water)

The stock AuPS25 NP suspension obtained in 0.5 mL absolute ethanol (denoted as AuPS25_EtOH) at 5 mg/mL was subjected to evaporation with no heat using the Thermo Savant SpeedVac SPD2030 (Thermo Fisher Scientific, Waltham, MA, USA) [54]. The dried pellet was resuspended in 1 mL of water (HyClone cell culture-grade endotoxin-free water (CGW or water)), vortexed for 30 s, and washed thrice by evaporation with no heat using the SpeedVac vacuum concentrator (Thermo Fisher Scientific, Waltham, MA, USA). The final pellet was resuspended in 0.5 mL of water (final concentration: 10 mg/mL), vortexed for 30 s, and stored at 4 °C in a sterile amber glass tube.

To achieve adequate dispersion in water, the AuPS25 NP (0.2 mg/mL) suspension was vortexed for 30 s and water-bath-sonicated for 0 s, 30 s, 60 s, 90 s, and 120 s at 40 kHz using a Branson 1800 Ultrasonic Cleaner (Branson Ultrasonics, Brookfield, CT, USA). The hydrodynamic diameters (*d*_H_) and polydispersity indices (PdI) were assessed using a Zetasizer Nano-ZS (Malvern Pananalytical, Inc., Sunnyvale, CA, USA) as previously described [58] at each time interval. The shortest sonication time that produced a stable and homogeneous dispersion was subsequently used for sample preparation before each characterization technique and risk assessment study. Additional details are provided in Supplementary Information S1, Supplementary Figure S1A,B, and Supplementary Table S1.

### 2.3. UV Aging of AuPS25 NPs

The AuPS25 NPs suspended in water after the removal of the ethanol were diluted to 0.2 mg/mL in 20 mL of water and added to a sterile glass beaker. The NPs were aged under a UV lamp using the Q-SUN Xe-1 Xenon Test chamber (Sonacme, Westlake, OH, USA) as detailed by Das et al. [45]. Briefly, the irradiance of the UV lamp was set at 0.55 W/m^2^/nm at 300–400 nm. The black standard temperature was set at 65 °C. A UV-transparent quartz Petri dish was used as a lid for the beaker to minimize attenuation of the UV light. This accelerated UV aging process is engineered to replicate the outdoor weathering of materials. Specifically, this method simulates the effects of six months of environmental exposure within 21 days, one year within 42 days, and ten years within 420 days [59,60]. The beaker was vigorously shaken once a day to ensure uniform aging. During the process, 5 mL of the suspension was collected at day 0 (denoted as AuPS25_0d), as well as on the 7th (AuPS25_7d), 14th (AuPS25_14d), and 21st (AuPS25_21d) days of UV aging, in sterile amber glass tubes.

### 2.4. Physicochemical and Morphological Characterization of AuPS25 NPs as a Function of UV Exposure Time

The size distribution and morphology of the AuPS25_0d, AuPS25_7d, AuPS25_14d, and AuPS25_21d NPs were assessed at accelerating voltages of 60, 150, and 400 kV using a transmission electron microscope (TEM, JEOL 2010 F) [54]. ImageJ software 1.53t was used to determine particle diameters and their size distribution from a sample set of over 180 individual particles as a function of UV exposure.

The *d*_H_, PdI, zeta potential (ζ), and conductivity (σ) of AuPS25 NPs at different UV exposure timepoints in water were assessed using a Zetasizer Nano-ZS [58].

The surface functional groups of AuPS25 NPs across UV exposure timepoints were measured using a Nicolet iS50 FTIR (Thermo Fisher Scientific, Waltham, MA, USA) with a Golden Gate high-temperature attachment (Specac, Orpington, UK) [45]. The ATR is a single-bounce monolithic diamond crystal with a 45° angle of incidence. The ATR crystal was heated to 45 °C. A 20 µL sample of the NP suspension was drop-casted onto the ATR crystal and allowed to dry. FT-IR spectra were obtained over a wavelength range of 600–4000 cm^−1^, at a resolution of 4 cm^−1^ and with an optical velocity of 0.1581, with 32 scans being acquired for each sample. The spectra were preprocessed by trimming data below 750 cm^−1^, baseline-corrected by Asymmetric Least Squares baseline correction (penalty term = 1 × 10^10^, asymmetry term = 0.0001), and normalized by dividing by the area (to account for differences in layer thickness) using the OriginPro and OMNIC 8.2.0.387 software.

The elemental composition of the AuPS25 NPs at different UV exposure timepoints was assessed using X-ray photoelectron spectroscopy (XPS, Thermo K-Alpha, Thermo Fisher Scientific, Waltham, MA, USA), comprising a K-alpha X-ray photoelectron spectrophotometer (Thermo Fisher Scientific, Waltham, MA, USA) with an Al Kα source (1486.6 eV) micro-focused monochromator [45]. To achieve an adequate sample thickness of over 400 µm, layers of NPs were drop-cast on the surface of copper holders. The base pressure was kept at approximately 1.01 × 10^−8^ mbar, and C1s was used as an internal energy reference with a binding energy value of 284.8 eV. Data analysis was performed using the CasaXPS 2.3.24 and XPS Peak fit 41 software.

### 2.5. Ethanol and Endotoxin Content and Microbiological Sterility Assessment of Un-Aged and Aged AuPS25 NPs

The ethanol content in un-aged and aged AuPS25 NPs was assessed using the MAK481 ethanol assay kit, following the supplier’s guidelines (Supplementary Information S2).

The endotoxin contamination of un-aged and aged AuPS25 NPs was assessed using the HEK-Blue™ LPS Detection Kit 2 (Invivogen, San Diego, CA, USA) according to the manufacturer’s instructions (Supplementary Information S3).

The microbiological sterility of un-aged and aged AuPS25 NPs was evaluated following the standard protocols established by the World Health Organization (WHO), as previously detailed [54] (Supplementary Information S3).

### 2.6. Oral Doses of AuPS25 NPs Used in Toxicity Studies

Due to a lack of accurate human exposure data, relevant environmental, water, and food concentrations for NPs are not well known. Previous exposure estimates (e.g., 5 g of NPs per week) were based on limited studies of NPs identified by microscopy/spectroscopy techniques, and are thus likely to be flawed. However, a small number of recent studies have employed Py-GC/MS to provide more accurate and complete quantification of total plastics in water and food. Although MNP levels found in drinking, surface, and ground water were relatively low (<1 µg/mL) [61,62], the amounts of plastics found in foods were much higher, ranging from about 10 µg/g in milk [63], 300 µg/g in rice [64], and 700 µg/g in pork [63], to 3 mg/g in sardines [13] and >10 mg/g in ground beef [63]. Since solid food comprises at least one-fourth of total oral intake, average MNP intake concentrations are thus likely to be as high as several hundred µg/mL. This is further supported by the finding of MNP levels up to 13 µg/mL in blood from healthy human volunteers [65]. In this study, we therefore employed relatively conservative target oral concentrations or TOCs of 4 and 40 µg/mL.

In order to achieve the desired TOCs of 40 and 4 µg/mL of AuPS25 NPs for toxicological assessments, the starting food model (i.e., water) concentrations were adjusted to account for two methodological variables: (1) the dilution of the final product of simulated digestion (digesta) prior to application to the SIE model in order to alleviate potential digesta toxicity due to malnutrition of cells, and (2) the greater density of AuPS25 NPs compared to pure PS NPs.

First, in order to avoid direct toxicity or nutrient deprivation, the final SI digesta of the AuPS25 NPs (as described below in Section 2.9 and Section 2.10) were diluted in cell culture media before being applied to the triculture epithelial model. The starting food model concentration was therefore adjusted (increased) by the dilution factor used in order to achieve a AuPS25 NP concentration in the triculture model that corresponds to the TOC. In pilot studies, we determined that the minimal dilution required to avoid direct toxic effects or reduction in critical nutrients caused by the replacement of media with the digesta was 12-fold. In this study, the final SI digesta of AuPS25 NPs from simulated digestions were therefore diluted by a factor of 12 in cell culture media before application to the triculture model, and the starting food model concentration was increased by a factor of 12 over the TOC to account for this dilution (as described below in Section 2.10).

A second adjustment was required to account for the substantially greater density of AuPS25 NPs compared to otherwise equivalent pure PS NPs due to the Au core. In order to reproduce the TOC for pure plastic NPs, the adjusted food model concentration (AFMC) of the AuPS25 NPs was increased by the ratio of the AuPS25 NP density to the corresponding pure PS NP density. The density of the AuPS25 NPs was determined from the diameter of the Au cores and the thickness of the PS shells, which were estimated from TEM images. For example, based on the TEM images, the average Au core diameter was 22 nm, and the average PS shell thickness was 4.5 nm. From this, we calculated that the total volume of AuPS25 NPs consisted of ~35.74% gold core (density 19.32 g/cm^3^) and ~64.26% PS shell (density 1.04 g/cm^3^), and that the average density of the AuPS25 particles was 10.63 g/cm^3^. Thus, in order to replicate a TOC of pure PS NPs of the same size, the AFMC must be increased by a factor of 10.63/1.04, or 7.57.

Taking both the dilution of digesta prior to application to cells and the density of the core–shell NP into account, the AFMC corresponding to a given TOC was calculated using the following equation:

(1) $$AFMC=TOC\times {Dil}_{Media}\times \frac{\rho_{Au-Plastic}}{\rho_{Plastic}}$$

where *AFMC* is the adjusted food model concentration, *TOC* is the target oral concentration, *Dil_Media_* is the dilution of the final digesta before application to small intestinal cells, *ρ_Au-Plastic_* is the density of the gold core–plastic shell NP (e.g., AuPS25), and *ρ_Plastic_* is the density of the plastic material of interest (e.g., PS).

Based on the formula, the equivalent AFMC doses for 40 and 4 µg/mL TOCs for AuPS25 NPs were calculated to be 3.633 mg/mL and 0.3633 mg/mL, respectively.

### 2.7. In Vitro Simulated Digestion of Un-Aged and Aged AuPS25 NPs in a Food Model (Water)

The in vitro simulated digestion was conducted using a 3-phase (oral, gastric, and SI) simulator, as previously detailed by DeLoid et al. [56,66]. Briefly, in the oral phase, water (control food model without NPs or blank digesta) and un-aged and aged AuPS25 NPs (AFMC: 3.633 mg/mL and 0.3633 mg/mL) in water were mixed 1:1 with pre-warmed (at 37 °C) simulated saliva containing mucin and other salts at a pH of 6.8 in amber glass tubes. The tubes were inverted manually for 15 s to complete the oral phase. To simulate the gastric phase of digestion, the resultant oral-phase digesta were then combined at a 1:1 ratio with pre-warmed simulated gastric fluid, comprising pepsin, hydrochloric acid (HCl), and sodium chloride (NaCl), and incubated in an orbital shaker at 200 rpm for 2 h. The gastric digesta were then mixed with bile salts, pancreatin (which includes a comprehensive set of pancreatic digestive enzymes), and additional salts, resulting in a dilution of the gastric digesta by a factor of three. The pH of this mixture was adjusted to 7.0 by adding sodium hydroxide (NaOH) to replicate small intestinal fluid. The SI digesta were then incubated in the orbital shaker at 37 °C and 200 rpm for 2 h.

### 2.8. Colloidal Characterization of SI Digesta of Food Model Containing Un-Aged and Aged AuPS25 NPs

A multi-angle laser diffraction (MALD) particle size analyzer (Mastersizer 3000, Malvern Panalytical, Sunnyvale, CA, USA) equipped with a wet dispersion unit (Hydro SV, Malvern Panalytical, Sunnyvale, CA, USA) and 633 and 466 nm laser sources was used to measure the volume-weighted particle size distributions (D [4,3]) of SI digesta of water containing un-aged and aged AuPS25 NPs [67]. The SI digesta of water containing 40 µg/mL TOC of un-aged and aged AuPS25 NPs were measured while stirring at 1800 rpm. The volume-weighted size distributions were averaged using the built-in software.

### 2.9. Preparation of In Vitro Transwell Triculture SIE Model

The transwell triculture SIE model was prepared as detailed previously [57,68]. Briefly, Caco-2 (Sigma-Aldrich, St. Louis, MO, USA) (a cell line derived from human colorectal adenocarcinoma, which resembles intestinal enterocytes upon maturation in culture) and HT29-MTX (Sigma-Aldrich, St. Louis, MO, USA) cells were cultured in 150 cm^2^ cell culture flasks (Corning, Corning, NY, USA) containing high-glucose DMEM (Thermo Fisher Scientific, Waltham, MA, USA) supplemented with 10% heat-inactivated fetal bovine serum (HI-FBS) (Sigma-Aldrich, St. Louis, MO, USA), a 10 mM HEPES buffer (Lonza, Basel, Switzerland), 100 IU/mL penicillin plus 100 μg/mL streptomycin (Corning, Corning, NY, USA), and non-essential amino acids (1/100 dilution of 100X solution) (Thermo Fisher Scientific, Waltham, MA, USA). Raji-B cells (Sigma-Aldrich, St. Louis, MO, USA) were cultured in RPMI 1640 media (Thermo Fisher Scientific, MA, USA) supplemented with 10% HI-FBS, 10 mM HEPES buffer, and 100 IU/mL penicillin plus 100 μg/mL streptomycin. Caco-2 and HT29-MTX cells were collected at passages 10–25 using TrypLE Express (Thermo Fisher Scientific, Waltham, MA, USA) and then resuspended in complete DMEM at a 3 × 10^5^ live cells/mL density for each cell type. These two cell suspensions were combined in a ratio of 3 parts Caco-2 cells to 1 part HT29-MTX cells. Following this, 1.5 mL of the cell mixture was added to the upper (apical) compartments of 6-well transwell plates (24 mm, polycarbonate membrane with an 8 µm pore size) (Corning, Corning, NY, USA), while 2.5 mL of complete DMEM was introduced into the lower (basolateral) compartments. The transwell plates were incubated at 37 °C and 5% CO_2_, with media changes taking place after 4 days and subsequently every other day until day 16. The media in the lower compartments were replaced on days 16 and 17 with 2.5 mL of a Raji-B cell suspension, which was harvested at passages 10–20 at a concentration of 1 × 10^6^ live cells/mL in a 1:1 mixture of complete DMEM and complete RPMI media.

Details on the physiological relevance of the triculture SIE model are provided in Supplementary Information S4.

### 2.10. Exposure of the SIE Model to the Digesta of the Food Model (Water) Containing Un-Aged and Aged AuPS25 NPs

The exposure of the triculture SIE model to the SI digesta of water (without NPs), un-aged and aged AuPS25 NPs in water, was performed on day 18, as stated earlier [57]. The SI digesta of water and test NPs (TOCs: 40 µg/mL and 4 µg/mL) were mixed with high-glucose DMEM without phenol red but supplemented with 10 mM HEPES buffer, 100 IU/mL penicillin, 100 μg/mL streptomycin, and non-essential amino acids at a 1:11 ratio. The apical media were replaced with 2.5 mL of media alone (untreated or negative control) or digesta–media mixtures, and the basolateral media were replaced with 2.5 mL of complete DMEM without phenol red but supplemented with 10% HI-FBS, 10 mM HEPES buffer, 100 IU/mL penicillin, 100 μg/mL streptomycin, and non-essential amino acids. The transwell plates were incubated at 37 °C and 5% CO_2_ for 24 h.

### 2.11. Toxicological Analysis

The reactive oxygen species (ROS) production, lactate dehydrogenase (LDH) release, trans-epithelial electrical resistance (TEER), and dextran permeability were assessed as described previously [57] (Supplementary Information S5).

### 2.12. Sample Collection and ICP-MS Analysis of Samples to Assess Uptake and Translocation of Un-Aged and Aged AuPS25 NPs in the SIE Model

The uptake and translocation of un-aged and aged AuPS25 NPs by/through intestinal enterocytes was quantified using ICP-MS [54]. Before TEER assessment, 2 mL of apical and basolateral fluids was collected in sterile amber glass tubes from each transwell plate, assigned to blank digesta and test AuPS25 NPs. After the dextran permeability assessment, the apical and basolateral compartments, designated as blank digesta and test AuPS25 NPs, were washed with 4 mL of PBS. Following washing and aspiration of PBS, 0.5 mL of 2X RIPA buffer was added to the apical chambers and incubated at RT for 10 min. Then, 2 mL of sterile DI water was added to the apical chamber and pipetted up and down, and a cell scraper was used to remove cells/debris from the insert membranes, which were ultimately collected in sterile amber glass tubes.

The AuPS25 NPs in apical and basolateral fluids, and cell lysates collected from toxicological assessments, were digested using aqua regia solution [54]. Briefly, 0.5 mL of the sample was added to 2.5 mL of aqua regia (3 parts of HCl to 1 part of HNO_3_) solution and incubated at RT for 12 h. The suspension was further diluted by adding 22 mL of DI water to bring the total acid concentration to below 10%. The samples were filtered using 0.22-micron cellulose filters prior to ICP-MS analysis. Analysis was performed using an Agilent 7850x ICP-MS (Agilent, Santa Clara, CA, USA) to quantify ^197^Au. For QC purposes, ^209^Bi was included as an internal standard, and a known Au concentration was analyzed after every 15 experimental samples. A one-minute wash with a 5 % HCl solution was performed before and after each sample read. The percentage of un-aged and aged NPs taken up by and translocated through epithelial cells was calculated using the following Equations (2) and (3):

(2) $$\%\;NP\;uptake=100\times \frac{Lysate\;NP\;concentration\times Lysate\;vol.}{Starting\;apical\;NP\;conentration\times Apical\;vol.}$$

(3) $$\%\;NP\;translocation=100\times \frac{Final\;basolateral\;NP\;concentration\times Basolateral\;vol.}{Starting\;apical\;NP\;conentration\times Apical\;vol.}$$

### 2.13. Assessment of Au Leaching During In Vitro Simulated Digestion of Un-Aged and Aged AuPS25 NPs

To investigate the potential leaching of Au from both un-aged and aged AuPS25 during the digestion process—an occurrence that could lead to the presence of Au ions in the AuPS25 NP digesta and thus affect toxicity results—the final SI digesta of aged and un-aged AuPS25 were filtered using a 3 kDa centrifugal filter (Nanosep 3K Omega) (Cytiva Life Sciences, Marlborough, MA, USA) [54]. The samples were centrifuged at room temperature at 15,000× *g* for 1 h. Au concentrations in both the unfiltered samples and the ultrafiltrates were quantified using an ICP-MS.

### 2.14. Statistical Analysis

Assessments of ethanol and endotoxin content and microbiological sterility were performed in quadruplicate for each treatment. Toxicological analysis, uptake, and translocation assessment were conducted with an N of 6 per treatment. Statistical analysis was performed using Prism 10.2.1 software (GraphPad Software Inc., San Diego, CA, USA). The results of DLS, toxicological assessments, and the uptake and translocation of NP treatments were analyzed using one-way ANOVA, followed by Dunnett’s multiple comparisons test.

## 3. Results and Discussion

### 3.1. UV Aging Impact on the Physicochemical and Morphological Properties of AuPS25

**Figure 2:**
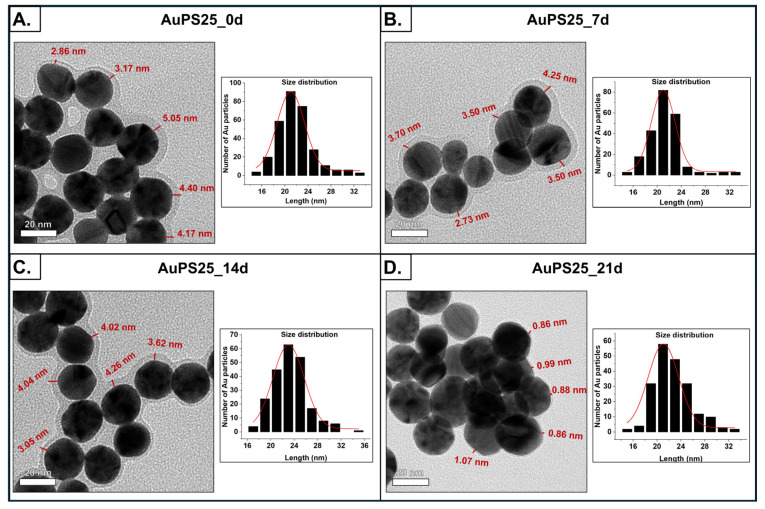
Morphological characterization of un-aged and aged AuPS25 NPs. TEM images and size distribution of (**A**) un-aged AuPS25 NPs, (**B**) 7-day-aged AuPS25 NPs, (**C**) 14-day-aged AuPS25 NPs, and (**D**) 21-day-aged AuPS25 NPs. The thickness of the PS shells is indicated in red in the TEM images of un-aged and aged AuPS25 NPs.

The surface morphology of the AuPS25 NPs was assessed using transmission electron microscopy (TEM) as a function of UV exposure time (0, 7, 14, and 21 days). The TEM images are shown in Figure 2A–D, revealing uniform spherical particles with dense centers corresponding to the Au cores. The Au cores had an average diameter of between 21 and 24 nm, irrespective of the aging period, suggesting that UV aging had no effect on Au core size. The low-density peripheral layer, corresponding to the PS shell, with a thickness between 3 and 4.5 nm, was evident in the TEM images corresponding to 0, 7, and 14 days (labeled as AuPS25_0d, AuPS25_7d, and AuPS25_14d). Although 7 and 14 days of UV aging had no effect on the thickness of the PS shell, a significant decrease in thickness, from ~4 nm to ~0.80 nm, was observed on the 21st day of UV aging, indicating that between the 14th and 21st day of weathering, the PS shell had begun to degrade and become attenuated as a result of the continued UV exposure. One goal of this work was to identify a timeframe in which UV aging significantly impacts the plastic shell without substantially attenuating the shell thickness (and potentially exposing the Au cores, which could directly interact with cells and confound toxicological studies). As the shell thickness was greatly attenuated on the 21st day, we elected to exclude AuPS25 samples aged beyond the 14th day from further toxicity investigations. Importantly, 14 days of UV aging in the Q-Sun Xenon test chamber approximates 60 days of outdoor weathering of PS NPs [45,59,60].

**Figure 3:**
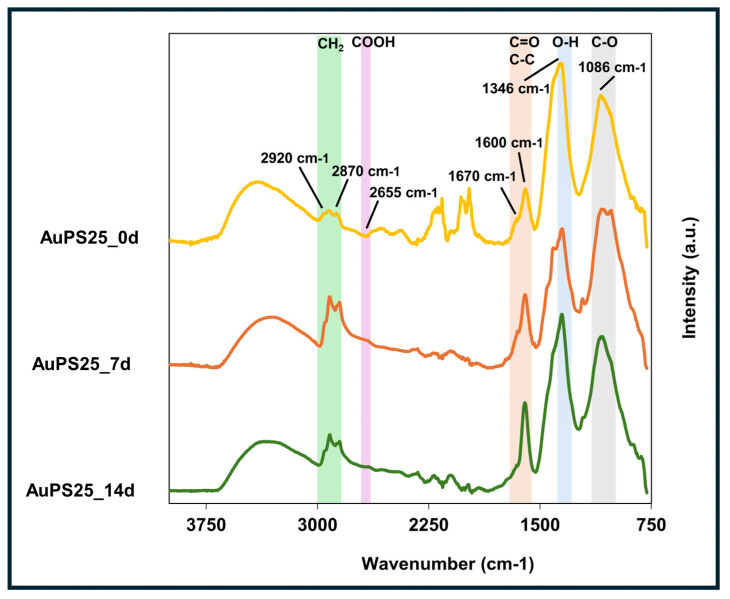
ATR-FT-IR spectra of un-aged, 7-day-aged, and 14-day-aged AuPS25 NPs.

FT-IR analysis was conducted to assess the effect of UV aging on functional groups of AuPS25 NPs as a function of exposure time (Figure 3). The major characteristic peaks at 2930, 2870, and 1600 wavenumbers, corresponding to pristine PS [69], were observed in un-aged AuPS25 NPs (0 days of UV exposure). The peak at 1600 cm^−1^ represents the C=C vinyl group, whereas the characteristic peaks at 2920 and 2850 cm^−1^ are related to CH_2_ asymmetric stretching and CH_2_ symmetric stretching, respectively. UV aging caused significant changes in the functional groups of AuPS25 NPs, indicative of photo-autooxidation. Specifically, after 7 and 14 days of UV aging, no aromatic C-H stretches (>3000 cm^−1^) were observed, suggesting the loss of aromatic moieties in the 7-day exposure timeframe. The formation of carboxylic groups after UV aging was evident through broad O-H stretch, O-H in-plane bending (1346 cm^−1^), C-O stretch (1086 cm^−1^), and a decrease in the carbonyl peak at 1670 cm^−1^, leaving only the peak at 1600 cm^−1^. Interestingly, the changes were not monotonic with time, and UV aging appeared to produce more acidic groups over time. Upon UV aging, enhanced alkyl C-H stretches and carboxylic acid overtone (2655 cm^−1^) were observed. The enhancement of carbonyl shoulders (1655, 1538 cm^−1^) suggests changes in the relative height of the carbonyl group. The peak with a maximum at 1346 cm^−1^ could be an O-H deformation in-plane, and the peak at 1086 cm^−1^ a C-O stretch. With the knowledge of an acidic proton, carbonyl, and C-O linkage, the UV aging induced the formation of carboxylic acid groups at 7 days of exposure and beyond was estimated. If the carboxylic acid were made on the alpha carbon of a monosubstituted aromatic ring, one would expect the carbonyl group to be blue-shifted towards 1750 cm^−1^. However, the lack of evidence of aromatic C-H stretches above 3000 cm^−1^ suggests that the carboxylic acid formed on aliphatic compounds.

**Figure 4:**
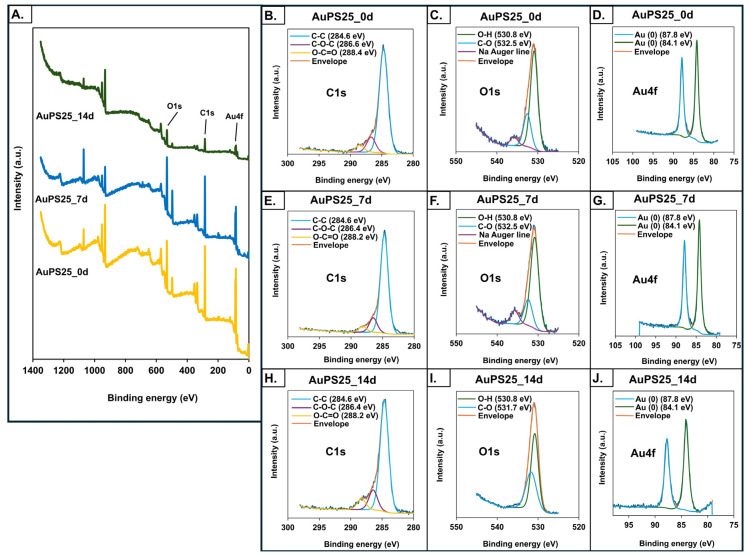
X-ray photoelectron spectroscopy (XPS) analysis of un-aged, 7-day-aged, and 14-day-aged AuPS25 NPs. XPS spectra of (**A**) un-aged and aged AuPS25 NPs; (**B**–**D**) carbon (C), oxygen (O), and gold (Au) spectra of un-aged AuPS25 NPs; (**E**–**G**) carbon (C), oxygen (O), and gold (Au) spectra of 7-day-aged AuPS25 NPs; (**H**–**J**) carbon (C), oxygen (O), and gold (Au) spectra of 14-day-aged AuPS25 NPs. Green and orange lines were indicating 'envelope' in (**B**–**J**).

XPS spectra to assess the elemental composition of un-aged and aged AuPS25 NPs (Figure 4A) depict three major binding energy lines for gold (Au4f_7/2_ and Au4f_5/2_), consistent with the Au cores, as well as single and double-bonded carbon, consistent with PS [54]. A decrease in C composition and an increase in O composition over the duration of UV aging were evident, as shown in Table 1.

In particular, the 5.9% increase in O content observed in AuPS25_14d suggests the formation of oxygen-containing functional groups. Notably, no alterations in the Au4f state were detected prior to or after UV aging. Although no significant change in chemical state was observed in the case of C1s before and after UV aging, a major shift in the O1s peaks from 532.6 eV to 531.7 eV after 14 days of aging was detected (Figure 4B–J), indicating the formation of carbonyl compounds, and suggesting photo-oxidation [39]. A significant decrease in the intensity of AuPS25_14d is probably due to the photo-oxidation of PS chains, leading to a loss of original non-polar C-H and C-C bonds and a decrease in overall surface area accessible for the analysis. The detected decrease in non-polar groups and increase in oxygen content on the surface of AuPS25 after 14 days of UV aging was supported by an increase in the negative charge as measured by zeta potential (as detailed below). These findings align with Hayek et al., who reported similar changes in peak intensity of polar functional groups, oxygen content, and zeta potential in UV-irradiated PS microspheres [70].

The *d*_H_, PdI, ζ, and σ of un-aged (AuPS25_EtOH, AuPS25_0d) and aged (AuPS25_7d and AuPS25_14d) NPs suspended in water are presented in Table 2 and Supplementary Figure S2. The hydrodynamic size of AuPS25_EtOH was 101.5 ± 3.28 nm, which increased significantly to 124.5 ± 1.34 nm when ethanol was removed and NPs were suspended in water, suggesting minor agglomeration of AuPS25 in water. Upon UV aging, the *d*_H_ of AuPS25_14d in water increased further, from 124.5 ± 1.34 nm to 131.63 ± 0.80 nm, suggesting additional agglomeration. The results showed no indication of particle fragmentation due to UV aging. The PdI for AuPS25_EtOH, un-aged, and 7- and 14-day-aged AuPS25 NPs ranged between 0.241 and 0.260, suggesting relatively monodisperse samples. As expected, the specific conductance measurement of un-aged and 7- and 14-day-aged AuPS25 NPs in water ranged between 0.045 and 0.059 mS/cm, which approximates the conductivity of pure distilled and deionized water. AuPS25_EtOH showed a ζ of +15.2 ± 0.75 mV because of the inherent polarity of the ethanol molecule. The ζ of AuPS25_0d decreased significantly from −9.2 ± 0.39 mV to −22.83 ± 1.36 mV and −24.06 ± 0.60 mV after 7 and 14 days of UV aging, respectively, suggesting enhanced electrostatic repulsion and dispersion. Further decreases in zeta potential upon UV aging are likely due to increased oxygen-containing functional groups, suggesting photooxidation. These observations align with Lui et al., Mao et al., and Xu et al., who reported that the zeta potential of PS NPs became increasingly negative with prolonged exposure to UV irradiation [71,72,73].

Endotoxin and microbiological sterility of AuPS25 NPs used in toxicity studies: In order to rule out the possibility of interference from residual ethanol or microbiological contamination of the PS NPs in toxicological assessments, we assessed the ethanol content, endotoxin concentration, and microbial sterility of AuPS25_0d and AuPS25_14d NPs. The ethanol content detected in un-aged (0.015%) and 14-day-aged AuPS25 NPs (0.003%) was significantly lower than the concentration of ethanol regarded as safe for toxicological analysis [74] (Supplementary Figure S3). Both un-aged and 14-day-aged AuPS25 NPs were sterile, and the endotoxin level was found to be below the detection limit of 0.017 EU/mL (Supplementary Information S3).

### 3.2. Leaching of Au During Digestion of Un-Aged and Aged AuPS25 NPs in Water

Analysis of the ultrafiltrates from the final SI phase of AuPS25 digesta using ICP-MS showed no negligible leaching of Au ions from either aged or un-aged AuPS25 particles. Specifically, only 0.05% and 0.07% of Au was leached from the un-aged and aged AuPS25 NPs, respectively. Given that the samples were further diluted 1:11 in culture media before application to the triculture SIE model, the amount of Au that would reach the cells is negligible.

These findings are consistent with those of our previous study, in which we reported that leaching of gold from un-aged AuPS25 NPs in SI digesta was approximately 0.03% [54]. While studies examining the toxicity of Au in mammalian systems are limited, no adverse effects from Au have been observed in *E. coli* at concentrations below 15 µM [75]. Thus, it is unlikely that the small amount of Au leached from AuPS25 contributed to any toxicity associated with it.

### 3.3. Agglomeration Potential of Un-Aged and Aged AuPS25 NPs in SI Digesta

The particle size distribution of the SI digesta of water containing un-aged and aged AuPS25 NPs of 40 µg/mL TOC is presented in Supplementary Figure S4. Notably, the blank digesta and SI digesta with un-aged and aged AuPS25 NPs contained a dominant major peak between 4.5 and 8 μm. The comparatively broader peak between 30 and 80 μm, as observed in SI digesta with un-aged AuPS25 NPs, suggested the interaction of digestive proteins in SI digesta (e.g., mucin, pepsin, pancreatic enzymes), forming agglomerates with un-aged AuPS25 NPs [67]. The agglomeration could be attributed to the van der Waals attraction, as evidenced by the −9 mV zeta potential of un-aged AuPS25 NPs. On the contrary, SI digesta with aged AuPS25 NPs showed comparatively lower agglomeration, which could be due to the enhanced electrostatic repulsion between −24 mV charged aged AuPS25 NPs and typically negatively charged digestive proteins in SI digesta.

### 3.4. Toxicological Assessment of Ingested Un-Aged and Aged AuPS25 NPs in the SIE

**Figure 5:**
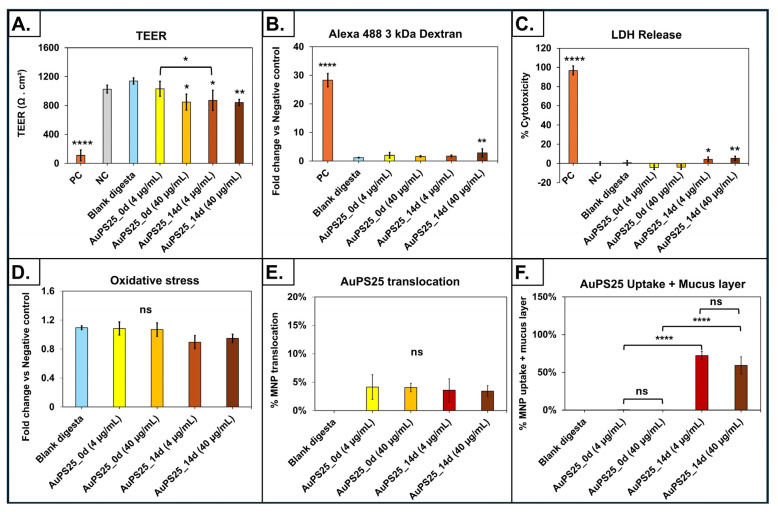
Assessment of toxicity, uptake, and translocation in an in vitro triculture small intestinal epithelium model exposed to SI digesta of un-aged and 14-day-aged AuPS25 in a food model (water). (**A**) TEER after 24 h exposure; (**B**) fold change in apparent permeability coefficient (*P*_app_) assessed with fluorescent-labeled 3 KDa dextran; (**C**) percent cytotoxicity (percent of LDH release relative to that of lysed control cells) after 24 h exposure; (**D**) reactive oxygen species (ROS, H_2_O_2_ equivalent concentrations) in apical fluid; (**E**) AuPS25 translocation through the intestinal epithelium; (**F**) AuPS25 uptake (+ mucus fraction of cell lysate) by intestinal epithelium. ns = non-significant; * = *p* < 0.05, ** *p* < 0.01 **** *p* < 0.001. Untreated cells that were exposed to 2X RIPA buffer to induce cell lysis were designated as the positive control (PC).

The digesta of aged AuPS25 NPs at 4 µg/mL TOC induced a significant (*p* < 0.05) decrease in TEER in comparison to the blank digesta (water, no NPs) and un-aged AuPS25 NPs of 4 µg/mL (Figure 5A). Specifically, when compared to the blank digesta, 4 µg/mL of AuPS25_14d NPs reduced TEER by 30.7% (*p* < 0.05), while 4 µg/mL of un-aged AuPS25 NPs had no impact. Furthermore, AuPS25_14d NPs at 4 µg/mL reduced (*p* < 0.05) TEER by 18.4% when compared to the un-aged AuPS25 NPs at 4 µg/mL.

Conversely, both the un-aged and 14-day-aged AuPS25 NPs at 40 µg/mL TOC reduced TEER by 34.4% and 35.4% (*p* < 0.05), respectively, when compared to blank digesta. AuPS25_14d at 40 µg/mL showed no significant (*p* > 0.05) difference in TEER when compared to un-aged AuPS25 of 40 µg/mL. These findings suggest that the effect of UV aging on impairment of epithelial barrier and tight junction integrity was only evident at the lower dose (i.e., 4 µg/mL of TOCs) of PS NPs.

Permeability of the SIE to fluorescently labeled 3kDa dextran, which indicates the integrity of the epithelial layer barrier function, was significantly (*p* < 0.05) increased after 24 h of exposure to the digesta of particularly aged AuPS25 NPs at 40 µg/mL TOC (Figure 5B). Specifically, not 4 µg/mL but only 40 µg/mL of aged AuPS25 NPs showed an increase (by 2.36-fold or 136%) in the apparent permeability coefficient (*P*_app_) in the SIE when compared to the blank digesta (water, no NPs), indicating dose-dependency. Further, a significant increase of 1.72-fold or 72.5% in the *P*_app_ of SIE was observed in the case of 40 µg/mL of aged AuPS25 NPs when compared to 40 µg/mL of the un-aged counterparts. Our findings overall suggest an impact of UV aging and concentration of PS NPs on cellular barrier integrity.

Unlike un-aged AuPS25 NPs at 4 and 40 µg/mL TOCs, exposure to 14-day-aged AuPS25 NPs at both 4 and 40 µg/mL TOCs showed significant (*p* < 0.05) cytotoxicity when compared to blank digesta (Figure 5C). Specifically, exposure to aged AuPS25 NPs at 4 and 40 µg/mL TOCs resulted in 4.37% and 5.42% increases in cytotoxicity, respectively, compared to blank digesta (water, no NPs).

In addition, the digesta of both un-aged and aged AuPS25 NPs of 4 and 40 µg/mL TOCs demonstrated no effect on ROS production when compared to the blank digesta (Figure 5D). This observation suggested that, irrespective of UV aging, ingested PS NPs at the tested TOCs do not impose oxidative stress in the SIE.

Collectively, these findings indicate that photo-aged AuPS25 NPs show significant cytotoxicity and disruption of cellular barrier integrity relative to un-aged particles, which is likely due to alteration in the physicochemical properties as a function of UV aging. Banerjee et al. previously reported that the surface properties of MNPs, including zeta potential, functional groups, and carbon/oxygen content, play a pivotal role in their cellular and molecular interactions, resulting in cellular toxicity [76]. Moreover, Eliane et al. showed that photoaged PS microspheres reduced cellular barrier integrity, consistent with our observations. They also reported that photo-oxidized PS caused a reduction in nuclear size, alterations of the cytoskeleton, and disruption of cellular metabolism and FAK expression [70]. These mechanisms suggested that the increased polar groups on the surface of PS NPs enhance cellular interactions with the particles, causing an increased toxic response [70].

Our observations for un-aged PS NPs are consistent with those from our previous studies with pristine non-carboxylated 25 nm PS NPs tested at relatively lower TOCs of 8.33 and 21.83 µg/mL in an SIE model [30]. We reported no change in TEER, cytotoxicity, dextran permeability, and oxidative stress at either of the tested TOCs [30]. We have also reported un-aged AuPS25 NPs at 60 µg/mL to cause minimal toxicity after 24 h exposure in either healthy or Crohn’s SIE derived from human biopsy samples in a microfluidic intestine-on-a-chip model [54]. We previously conducted toxicological assessments of carboxyl-modified 25 nm PS NPs (considered to be more ecologically relevant than pristine non-carboxylated PS NPs) in the SIE model [30]. The carboxylated PS NPs did not induce any significant alterations in TEER nor exhibited cytotoxic effects in SIE across any of the tested TOCs (8.33 and 21.83 µg/mL), in contrast to aged AuPS25 NPs tested in this study [30]. Our FT-IR analysis indicated carboxylic acid formation on the 14-day-aged AuPS25 NPs surface. Similarly, carboxyl-modified 25 nm PS NPs have also been shown to possess carboxylic acid functionalities [30]. The observed discrepancy in toxicity assessments between the aged AuPS25 NPs and carboxylated PS NPs may be understood by examining their zeta potential values. The carboxylated PS NPs demonstrated a zeta potential of −66 mV [66], whereas the zeta potential of the 14-day-aged AuPS25 NPs, as observed here, was −24 mV. The substantially higher zeta potential of the carboxylated PS NPs might have contributed to their comparatively lower cytotoxic effects, as the elevated electrostatic repulsion may reduce interactions with cell membranes [77,78].

### 3.5. Assessment of AuPS25 NP Uptake and Translocation in the SIE Model Using ICP-MS

The uptake by and translocation across the SIE of un-aged and 14-day-aged AuPS25 NPs were assessed using ICP-MS.

No significant (*p* < 0.05) difference was observed between the translocation of either un-aged or aged AuPS25 NPs (Figure 5E). Specifically, the translocation across the intestinal enterocytes by the un-aged AuPS25 NPs of 4 and 40 µg/mL TOCs was 4.1% and 4%, respectively, while the translocation by aged AuPS25 NPs of 4 and 40 µg/mL was 3.5% and 3.4%, respectively. Similarly, we previously reported that only 1.15% of the carboxylated 25 nm PS NPs were translocated across the SIE [30], while other recent studies reported no translocation of pristine and carboxylated PS NPs across the SIE [79,80].

Finally, the uptake (+ mucus fraction of cell lysate) of aged AuPS25 NPs by the SIE was dramatically greater than that of un-aged AuPS25 NPs. Specifically, 72.2% and 59.2% of aged AuPS25 NPs applied to the SIE were accumulated at 4 µg/mL and 40 µg/mL TOCs, respectively. No significant difference was, however, observed between the uptake rate of AuPS25 NPs at 4 µg/mL and 40 µg/mL TOCs. Notably, only 0.3% and 0.2% of un-aged AuPS25 were taken up at 4 and 40 µg/mL TOCs, respectively (Figure 5F).

Overall, these findings demonstrate that UV-aged PS NPs are much more readily absorbed by SIE than un-aged PS NPs, likely due to chemical modifications on the surface of the PS NPs induced during the UV aging processes and less agglomeration potential in SI digesta, as observed through MALD. In contrast, un-aged PS NPs demonstrated a propensity for higher levels of agglomeration within the SI digesta. This agglomerative behavior could substantially hinder their uptake by the SIE. Interestingly, studies have previously shown that photo-aged PS MNPs promote cell uptake by increasing cell membrane permeability [40,81]. Similarly, the aforementioned data on cellular permeability are confirmed herein.

Notably, elevated cellular uptake exhibited by aged AuPS25 NPs could be facilitated by both passive diffusion and active endocytic mechanisms. We previously reported that carboxylated 25 nm PS NP agglomerates cross the enterocyte plasma membrane independent of the endosomal compartment, indicating diffusion-based uptake, while other agglomerates were enclosed within actin shells, suggesting active (energy-dependent) uptake by either phagocytosis or micropinocytosis [30]. Additionally, a significant number of agglomerates were observed free within the cytoplasm, with notable localization within the nuclei [30]. In a further, in-depth mechanistic study employing specific uptake pathways and metabolic inhibitors, we determined that uptake of 25 nm PS NPs in the triculture SIE model occurred via both passive diffusion and active endocytic mechanisms, including phagocytosis, clathrin-mediated endocytosis (CME), and fast endophilin-mediated endocytosis (FEME) [66]. These results are consistent with additional findings on PS NPs’ uptake mechanisms in a primary human organoid-based intestine-on-a-chip SIE model [54].

Interestingly, we reported earlier that only 1.4% of carboxylated 25 nm PS NPs at 8.3 µg/mL TOCs were taken up by the SIE [30], while in this study, we found the uptake of aged AuPS25 NPs of 4 and 40 µg/mL to be over 72.2% and 59.2%, respectively. This discrepancy could again be justified based on the significant difference in zeta potential between carboxylated 25 nm PS NPs (−66 mV) and the aged AuPS25 NPs (−24 mV). However, a comparatively high percentage of 14-day-aged AuPS25 NPs in the cell lysate and mucus compartment is somewhat surprising. While this could represent greater cellular uptake, because translocation of AuPS25_14d NPs was not significantly different than that of AuPS25_0d NPs, it is likely that most of the AuPS25_14d in the lysate and mucus compartment was in fact located within the apical mucus rather than the cells [82]. A number of studies have previously reported similar uptake of PS NPs by intestinal epithelium [79,83]. For instance, recently, Marcellus et al. quantified 89% and 51% of 50 nm and 500 nm PS NPs, respectively, to be taken up by enterocytes and reported less than 1% of the PS NPs of either size to translocate across the SIE [79]. However, further studies will be required to determine if aged PS NPs are sequestered within the mucus, and if proven, the chemical interactions responsible for their apparent affinity.

This study also highlights the exquisite utility of ICP-MS for the quantification of gold-core tracer PS NPs. We determined the limit of detection (LOD) of Au of AuPS25 NPs in water, apical fluid (1 part of SI digesta + 11 parts of complete DMEM without FBS), basolateral fluid (complete DMEM with FBS), and cell lysates (RIPA buffer + media) to be 0.13, 0.03, 0.05, and 0.05 ppb, respectively. The limit of quantification (LOQ) for Au in water, apical and basolateral fluid, and cell lysates was found to be 0.71, 0.24, 0.41, and 0.4 ppb, respectively. Notably, the recovery rates for free Au spiked into AuPS25 NPs in water, apical and basolateral fluids, and cell lysates ranged from 95% to 99%. Current methodologies for MNP quantification predominantly employ techniques such as micro-Raman spectroscopy, micro-FT-IR, and LD-IR [47,84,85]. These microscopy- and spectroscopy-based particle identification and counting techniques are inherently limited to sizes above the resolution limit of the microscope and fail to detect particles in the nanoscale size range, which are likely to be the most bioactive and potentially hazardous. As such, they cannot provide accurate total mass concentrations of MNPs/plastics and are thus of limited utility. Conversely, Py-GCMS, a rapidly emerging analytical technique, has shown promising results, yet discrepancies remain apparent when quantifying MNPs in simplistic (such as water) versus complex biological matrices [86,87,88]. For example, Ccanccapa-Cartagena et al. quantified PS MNPs in tap water ranging from 0.86 to 2.57 ng/L [89]. However, Rauert et al. raised concerns about the applicability of Py-GCMS for lower concentrations of PS, PE, PET, and PVC due to issues of matrix suppression, interferences, and background contamination, notably in complex biological media, with limitations evident at concentrations below 1 µg/mL [90]. Another critical limitation of the Py-GCMS approach stems from the lack of standardized reference materials [91], leading to substantial over-estimation of PS concentrations—reported by Brits et al. to range from 1300% to 2600%—resulting from discrepancies between the polymer characteristics of calibration standards and those found in environmental samples [88]. Unlike ICP-MS, which offers greater sensitivity, Py-GCMS demands exhaustive sample preparation and stringent experimental conditions, rendering it less feasible for large-scale analysis [85]. Overall, these findings underscore the superior capability of ICP-MS in MNP quantification, prompting and indicating a need for further investigation into the synthesis of environmentally relevant ICP-MS-traceable gold-core plastic shell particles for enhanced biokinetic assessments.

## 4. Conclusions

UV aging profoundly alters the physicochemical properties of polystyrene nanoplastics, driving photo-oxidation, increased cytotoxicity, disruption of epithelial barriers, and enhanced uptake by intestinal enterocytes. By leveraging ICP-MS-traceable core–shell particles, we demonstrate nanoplastic detection at environmentally relevant concentrations and establish a robust framework for quantification using a super-sensitive analytical approach such as ICP-MS.

These findings emphasize the need to incorporate aged and environmentally relevant micro-nanoplastics into risk assessment, ensuring that toxicological evaluations reflect real-world exposures and better inform public health decision-making for this emerging contaminant.

## Figures and Tables

**Table 1:** The surface elemental composition of un-aged, 7-day-aged, and 14-day-aged AuPS25 NPs.

Elemental Composition				
Samples	C (%)	O (%)	Au (%)	Other (%)
AuPS25_0d	62.6	21.4	3.9	12.1
AuPS25_7d	60.7	22.4	2.6	13.3
AuPS25_14d	53.4	27.3	2.5	16.8

**Table 2:** Colloidal characterization of un-aged and aged AuPS25 samples. *d*_H_: intensity-weighted mean hydrodynamic diameter; *PdI*: polydispersity index; *ζ*: zeta potential; *σ*: specific conductance. Different letters indicate a significant difference (*p* < 0.05).

Sample	*d*_H_ (nm)	PdI	ζ (mV)	Σ (mS/cm)
AuPS25_EtOH	101.5 ± 3.28 ^a^	0.245 ± 0.008 ^a,b^	+15.2 ± 0.75 ^a^	0.00425 ± 0.00003 ^a^
AuPS25_0d	124.5 ± 1.34 ^b^	0.260 ± 0.008 ^b^	−9.2 ± 0.39 ^b^	0.049833 ± 0.0003 ^b^
AuPS25_7d	129.44 ± 3.44 ^b^	0.245 ± 0.006 ^a,b^	−22.83 ± 1.36 ^c^	0.059 ± 0.00065 ^c^
AuPS25_14d	131.63 ± 0.80 ^c^	0.241 ± 0.006 ^a^	−24.06 ± 0.60 ^d^	0.05693 ± 0.0017 ^d^
AuPS25_21d	145.5 ± 5.21 ^d^	0.242 ± 0.001 ^a^	−25.36 ± 0.81 ^d,e^	0.04547 ± 0.00025 ^e^

## Data Availability

The raw data represented in the results and supporting the conclusions of this article will be made available by the authors upon request.

## Supplementary Materials

The following supporting information can be downloaded at https://www.mdpi.com/article/10.3390/toxics13110939/s1: Supplementary Figure S1. Intensity-based size distribution of A. AuPS25 NPs in water at varying durations of water-bath sonication and B. 14-day-aged AuPS25 NPs on the 0th, 90th, and 180th days of storage at 4 °C; Supplementary Figure S2. Intensity-based size distribution of un-aged and aged AuPS25 NPs; Supplementary Figure S3. Ethanol content assessment of un-aged and 14-day-aged AuPS25 NPs; Supplementary Figure S4. Volume-weighted size distributions of final small intestinal digesta of water containing aged and un-aged AuPS25 NPs; Supplementary Table S1. Colloidal characterization of AuPS25 samples in water at varying durations of water-bath sonication. *d*_H_: intensity-weighted mean hydrodynamic diameter; *PdI*: polydispersity index. Different letters indicate a significant difference (*p* < 0.05); Supplementary Table S2. Colloidal stability of 14-day-aged AuPS25 samples on the 0th, 90th, and 180th days of storage at 4 °C. *d*_H_: intensity-weighted mean hydrodynamic diameter; *PdI*: polydispersity index; *ζ*: zeta potential; *σ*: specific conductance. Different letters indicate a significant difference (*p*  <  0.05). References [92,93,94,95,96,97,98,99,100,101,102,103,104,105,106] are cited in the supplementary materials.
