# Supplementary material for: Impact of UV Aging on the Toxicity and Bioavailability of Inductively Coupled Plasma Mass Spectrometry (ICP-MS)-Traceable Core–Shell Polystyrene Nanoplastics in an In Vitro Triculture Small Intestinal Epithelium Model
Source: Toxics. 2025 Oct 30;13(11):939. doi: 10.3390/toxics13110939 (PMC12656232; doi:10.3390/toxics13110939)
Supplement: Supplementary file 1 [file toxics-13-00939-s001.zip › toxics-3920437-supplementary.pdf]

Impact of UV Aging on the Toxicity and Bioavailability of Inductively Coupled Plasma Mass Spectrometry (ICP-MS)-Traceable Core–Shell Polystyrene Nanoplastics in an In Vitro Triculture Small Intestinal Epithelium Model

Satwik Majumder [1], Lila Bazina [1], Glen DeLoid [1], Alvaro G. Garcia [2], Nubia Zuverza-Mena [2], Jakub Konkol [3], George Tsilomelekis [3], Michael Verzi [4], Hao Zhu [5], Jason C. White [2] and Philip Demokritou [1,*]

[1] Nanoscience and Advanced Materials Center, Environmental and Occupational Health Sciences Institute (EOHSI), Rutgers Biomedical Health Sciences, Rutgers University, Piscataway, NJ 08854, USA; sm3397@eohsi.rutgers.edu (S.M.); lb948@gsbs.rutgers.edu (L.B.); gd424@eohsi.rutgers.edu (G.D.)

[2] Department of Analytical Chemistry, The Connecticut Agricultural Experiment Station, New Haven, CT 06511, USA; alvaro.g.garcia@ct.gov (A.G.G.); nubia.zuverza@ct.gov (N.Z.-M.); jason.white@ct.gov (J.C.W.)

[3] Department of Chemical and Biochemical Engineering, Rutgers University, 98 Brett Road, Piscataway, NJ 08854, USA; jak496@soe.rutgers.edu (J.K.); gt241@soe.rutgers.edu (G.T.)

[4] Department of Genetics, Rutgers University, Piscataway, NJ 08854, USA; mv347@rutgers.edu

[5] Center for Biomedical Informatics and Genomics, School of Medicine, Tulane University, New Orleans, LA 70112, USA; hzhu10@tulane.edu

* Correspondence: philip.demokritou@rutgers.edu; Tel.: +1-848-445-0155

Supplementary information 1: Dispersion of AuPS25 NPs in water

The dispersion characteristics of NPs within food matrices are critical for risk assessment, as they significantly influence the stability, concentration, and bioavailability of these particles in biological systems. The AuPS25 NP suspension was vortexed for 30 sec, followed by varying durations of water-bath sonication. Subsequently, hydrodynamic size measurements were taken using DLS **(Supplementary Figure S1A and Supplementary Table S1)**. The initial d_H value for AuPS25 NPs, prior to sonication, was recorded at 243.75±9.54 nm, indicating the presence of agglomerated particles. Notably, sonication for 30 seconds resulted in a significant reduction in the d_H value to 123.95±1.37 nm. However, prolonging the sonication duration beyond this point correlated with an incremental increase in d_H values, signifying the re-agglomeration of particles. Overall, our findings suggest that a 30-second sonication period is optimal for preserving the stability and homogeneity of AuPS25 NPs in water. The PdI for AuPS25 NPs across various water-bath sonication durations exhibited values ranging from 0.260 to 0.300. Notably, at the 30 sec mark of sonication, the PdI was recorded as 0.260, indicative of a predominantly monodisperse system and reflecting a narrow particle size distribution. In contrast, the PdI values for AuPS25 NPs without sonication and after 120 sec of sonication approached 0.300, which is at the threshold between a mostly monodisperse and slightly polydisperse classification. This increase in PdI suggests a broader size distribution, potentially affecting the NP stability and dispersion uniformity.

The colloidal stability of 14-day-aged AuPS25 NPs was examined after 90 and 180 days of storage at 4 °C by DLS. Given that AuPS25_14d NPs were designated for use in risk assessment studies, it is imperative that the sample demonstrates sustained stability within the food model over time. The findings indicate no major changes in the d_H, PdI, ζ, and σ values, suggesting that the samples remain stable in water throughout the storage period **(Supplementary Figure S1B** and **Supplementary Table S2)**.

Supplementary information 2: Ethanol content assessment

The ethanol content in un-aged and aged AuPS25 NPs was assessed using the MAK481 ethanol assay kit, following the supplier's guidelines. A total of 90 μL of the working reagent (prepared as suggested by the supplier) was added to 10 μL of the NP suspension (working concentration: 1 mg/mL) prior to being transferred to a 96-well plate. The plate was incubated for 30 min at room temperature (RT). After incubation, 100 μL of the Stop reagent was added to each well. Incremental concentrations (0.5%, 0.1%, 0.03%, and 0.06%) from 1% ethanol were used to prepare a standard curve. The absorbance was read at 565 nm using the SpectraMax M5 microplate reader (Molecular Devices, CA, USA). The slope from the standard curve was determined using linear regression fitting. The ethanol content, as a percentage, was calculated using Equation (S1), as follows:

$$\%\ \text{Ethanol} = DF \times ((OD_{NPs} - OD_{blank}) \div \text{Slope}) \qquad \text{(S1)}$$

where DF is the dilution factor, OD_{NPs} is the optical density of un-aged and aged AuPS25 NPs at 565 nm, and OD_{blank} is the optical density of blank (0% ethanol) at 565 nm.

To disregard the interference of ethanol and microbiological contamination in toxicological assessments, we verified the ethanol content, endotoxin presence, and microbiological sterility of AuPS25_0d and AuPS25_14d NPs. Assessing ethanol content is important, as earlier studies have reported DNA damage and a negative impact on Caco-2 cell viability at ethanol concentrations above 3% [1]. In this study, a trace amount of ethanol was detected in both the un-aged (0.015%) and 14-day-aged (0.003%) NPs **(Supplementary Figure S3),** which, however, was significantly lower than the concentration of ethanol regarded as safe for toxicological analysis [2].

Supplementary information 3: Endotoxin and microbiological sterility assessment

The endotoxin contamination of un-aged and aged AuPS25 NPs was assessed using the HEK-Blue™ LPS Detection Kit 2 (Invivogen, CA, USA) according to the manufacturer's instructions. Briefly, HEK-Blue™-4 cells were cultured in Dulbecco's Modified Eagle Medium (DMEM) without phenol red (Thermo-Fisher Scientific, MA, USA) and supplemented with 10% heat-inactivated ultra-low-endotoxin fetal bovine serum (HI-FBS) (Sigma-Aldrich, MO, USA) and 2 mM L-alanyl-L-glutamine (Corning, NY, USA), along with 100 U/mL penicillin and 100 µg/mL streptomycin (Thermo Fisher Scientific, MA, USA), 100 µg/mL Normocin, and the selected antibiotics provided in the kit. A 20 µL suspension of un-aged and aged AuPS25 NPs at a concentration of 0.1 mg/mL was dispensed into triplicate wells of a tissue culture-treated 96-well plate. Endotoxin standards ranging from 0.01 to 1 EU/mL were prepared using endotoxin derived from *Escherichia coli* serotype 055, provided in the kit, and placed in designated wells. Subsequently, a HEK-Blue™-4 cell suspension containing 48,000 cells in 160 µL was added to both sample and standard wells. The plate was incubated for 20 h at 37 °C in a 5% CO_2 atmosphere. After incubation, 40 µL of supernatant from each well was transferred to a detection plate, and 160 µL of Quanti-Blue (QB) reagent was added to each well. The plate was then incubated at 37°C for 2 h. Absorbance was measured at 620 nm, and the endotoxin concentrations were determined using the standard curve generated from the endotoxin standard wells.

The microbiological sterility of un-aged and aged AuPS25 NPs was evaluated following the standard protocols established by the World Health Organization (WHO) [3, 4]. In summary, a 1 mL sample of a 0.1 mg/mL suspension of un-aged and aged AuPS25 was combined with 10 mL of fluid thioglycolate medium, adjusted to a pH of 6.9–7.3, and incubated at 37 °C for 14 days. The mixture was examined daily for signs of bacterial growth, and assessments for bacterial and fungal contamination were conducted each day using a pour plate method with both potato dextrose agar and plate count agar.

The evaluation of sterility and endotoxin levels in NPs is paramount in toxicological studies, as contamination has been documented to compromise data integrity and lead to potential misinterpretations of immune-safety outcomes [5, 6]. According to the Food and Drug Administration (FDA), European Pharmacopeia (EP), and United States Pharmacopeia (USP), the acceptable limit for endotoxin is 0.5 EU/mL [7]. In this study, both un-aged and aged NPs were microbiologically sterile, and the endotoxin level was found to be below the detection limit of 0.017 EU/mL.

Supplementary information 4: Physiological relevance of the triculture SIE model

Most intestinal model employs Caco-2 (immortal human colonic epithelial) cells, which, after 2-3 weeks of culture, differentiate into cells with markers and morphological characteristics similar to those of small intestinal epithelial (SIE) enterocytes [8, 9]. However, the SIE is more complex, and to emulate this structure, several modifications are necessary. As the intestinal mucosa is protected by a layer of mucus produced by goblet and submucosal glands, we co-cultured Caco-2 cells with HT29-MTX cells, an immortal human cell line that resembles intestinal goblet cells and secretes mucus [8, 10, 11]. Lastly, in the Peyer's patch, microfold or M-cells are present that engulf and translocate samples from the intestinal lumen to the lymphocytes located in the submucosa beneath, ensuring ongoing monitoring of the antigens present in the intestinal contents [12]. To achieve this, we added Raji B cells (a human B lymphocyte) on the basolateral compartment of the transwell system to induce factors that differentiate Caco-2 cells into cells resembling M-cells [13-16]. Specifically, in this study, we added Raji B cells on the 16th and 17th day, right before the NP exposure (on day 18). Overall, this model includes cells with morphology and markers

consistent with the three primary cells of the SIE—enterocytes, goblet cells, and M-cells—and represents a reasonably realistic hybrid model of the complete SIE [16-18].

Supplementary information 5: Toxicological analysis

ROS production (oxidative stress) was assessed using the OxiSelect in vitro assay kit (Cell Biolabs, CA, USA) according to the manufacturer's protocol with minor adjustments [19]. The 1X Catalyst and DCFH solution (final reaction mixture) was prepared as guided by the manufacturer. After 6 h of the 24 h incubation, 150 μL of apical fluid was collected in sterile Eppendorf tubes from the transwell assigned to the NC, water (blank digesta), and test NPs. The tubes were centrifuged at 5000 × g for 5 min. To prepare standards, a fivefold serial dilution in sterile water was performed with 20 μM H_2O_2 solution. Further, 50 μL of NC, blank digesta, test NPs, and H_2O_2 standards were dispensed into a black-walled, clear-bottom 96-well plate. A total of 50 μL of the catalyst solution was added to each well, and the plate was incubated for 5 min at RT. Then, 100 μL of the final reaction mixture was added to each well, and the plate was incubated for 30 min at RT. Fluorescence was measured at 480/530 nm (excitation/emission) using a microplate reader, and equivalent μM H_2O_2 concentrations in test NPs were determined from a standard curve generated from H_2O_2 standards. The data were represented as fold-change vs. NC.

The release of LDH was measured using a Pierce LDH assay kit (Sigma-Aldrich, St., MO, USA) following the manufacturer's guidelines with certain modifications [19]. Briefly, 150 μL of apical media from one of the two plates assigned to the untreated control (NC) was replaced with 150 μL of 2X RIPA buffer (Thermo Fisher Scientific, MA, USA) 45 min before the 24 h exposure to create a positive control (PC). After 24 h exposure, 150 μL of apical fluid was collected in sterile Eppendorf tubes from each transwell designated for PC, NC, blank digesta, and test NPs and centrifuged at 10,000 × g for 5 min. A 50 μL sample from the centrifuged apical fluids was dispensed in a black-walled, clear-bottom 96-well plate (BD Biosciences, NJ, USA), to which 50 μL of reaction mixture, prepared according to the manufacturer's instructions, was added. The plate was incubated in the dark at RT for 30 min. Further, 50 μL of stop solution was added to each well, and absorbance was measured at 680 (A680) and 490 (A490) nm using a SpectraMax M5 microplate reader. Background corrected absorbance, A, was calculated for each well by subtracting A_{680} from A_{490}. The percentage cytotoxicity for each treatment well was calculated using Equation (S2):

$$\% \text{ Cytotoxicity} = (A_{NPs} - A_{NC} \div A_{PC} - A_{NC}) \times 100 \qquad \text{(S2)}$$

where ANPs is the absorbance of un-aged and aged AuPS25 NPs, ANC is the absorbance of the negative control, and APC is the absorbance of the positive control.

Trans-epithelial electrical resistance (TEER) was evaluated using the EVOM2 Epithelial V/Ω Meter with a chopstick Electrode Set (World Precision Instruments, FL, USA) as detailed earlier [19]. After LDH analysis, the apical and basolateral fluids were replaced with 3 mL of PBS. TEER measurements were taken in PC, NC, blank digesta, and test NPs to evaluate their impact on epithelial barriers and tight junction integrity. The TEER values were expressed in Ω/cm^2.

Dextran permeability was assessed using a fluorescently labeled dextran, Alexa Fluor 488 3 kDa (Thermo Fisher Scientific, MA, USA) [19]. A total of 1 mL of working solution per transwell insert to be tested, comprising 25 μg/mL of AF488 3 kDa dextran, was prepared in PBS. After TEER analysis, the transwells were washed twice with 3 mL PBS. Subsequently, 1 mL of working dextran solution was added to the apical and 2 mL of PBS to the basolateral compartments. The transwell plates were incubated at 37 °C and 5% CO_2 for 1 h. Then, 200 μL of basolateral fluid from plates assigned to PC, NC, blank digesta, and test NPs was collected, and the fluids were placed in a black-walled, clear-bottom 96-well plate. Fluorescence

was measured at Ex 495 nm/Em 519 nm for Alexa Fluor 488 3 kDa dextran using a SpectraMax M-5 microplate reader. Apparent permeability, P_{app} (cm/s), was calculated using Equation (S3):

$$Papp = (dQ \div dt) \div (A \times C0) = dQ \div (dt \times A \times C0) \quad \text{(S3)}$$

where dQ represents the quantity of dextran (in μg) present in the basolateral compartment, which is determined through fluorescence measurements and standard curves specific to each dextran; the variable dt indicates the duration (in seconds) from the moment the dextran is introduced into the apical compartment until it is measured in the basolateral compartment; A denotes the surface area of the transwell; and C_0 refers to the initial concentration of dextran in the apical compartment.

Supplementary Table

Sample	d_H (nm)	PdI
**AuPS25_0sec**	243.75±9.54^{c}	0.294±0.019^{a,b}
**AuPS25_30sec**	123.95±1.37^{a}	0.260±0.011^{a}
**AuPS25_60sec**	198.95±1.36^{b}	0.264±0.014^{a}
**AuPS25_90sec**	207.93±18.09^{b,c}	0.279±0.024^{a}
**AuPS25_120sec**	224.23±17.55^{c}	0.300±0.011^{b}

Supplementary Table S1. Colloidal characterization of AuPS25 samples in water at varying durations of water-bath sonication. d_H: intensity-weighted mean hydrodynamic diameter, *PdI*: polydispersity index. Different letters indicate a significant difference ($p < 0.05$).

Parameters	AuPS25_14d @ 0 day	AuPS25_14d @ 90 days	AuPS25_14d @ 180 days
**d_H (nm)**	131.63±0.80[a]	120±2.64[b]	122±1.39[b]
**PdI**	0.241±0.006[a]	0.246±0.005[a]	0.248±0.005[a]
**ζ (mV)**	-24.06±0.60[a]	-22.28±2.41[a]	-23.47±2.33[a]
**σ (mS/cm)**	0.05693±0.0017[a]	0.05275±0.0032[b]	0.05386±0.0025[c]

Supplementary Table S2. Colloidal stability of 14-day-aged AuPS25 samples on the 0th, 90th, and 180th days of storage at 4 °C. d_H: intensity-weighted mean hydrodynamic diameter, *PdI*: polydispersity index, *ζ:* zeta potential, *σ:* specific conductance. Different letters indicate a significant difference ($p < 0.05$).

Supplementary Figures

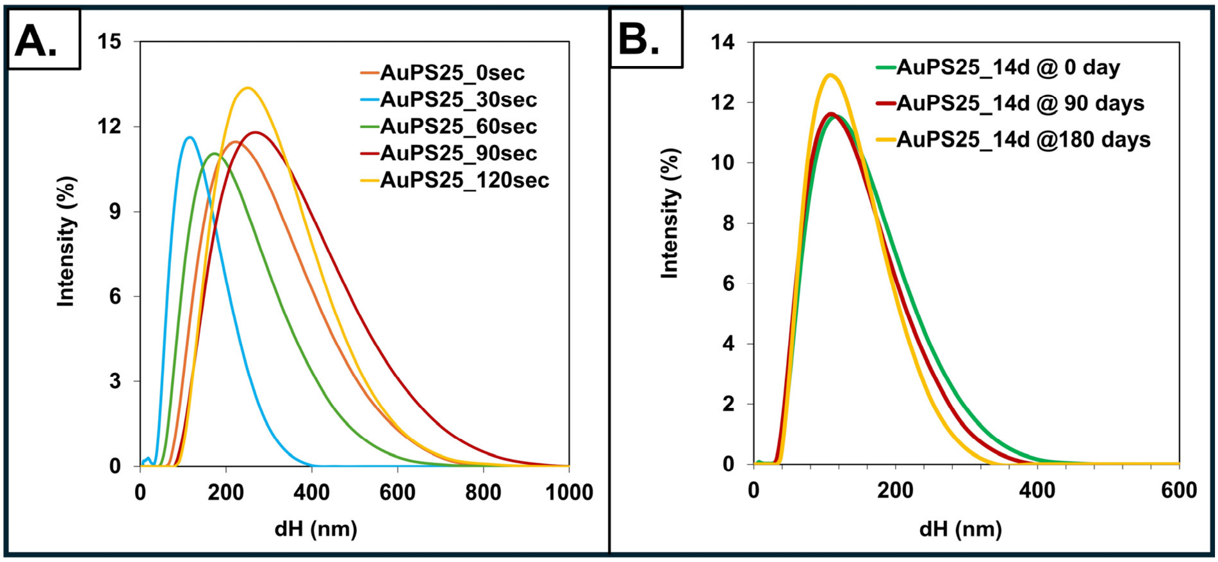

Supplementary Figure S1. Intensity-based size distribution of **A.** AuPS25 NPs in water at varying durations of water-bath sonication and **B.** 14-day-aged AuPS25 NPs on the 0th, 90th, and 180th days of storage at 4 °C.

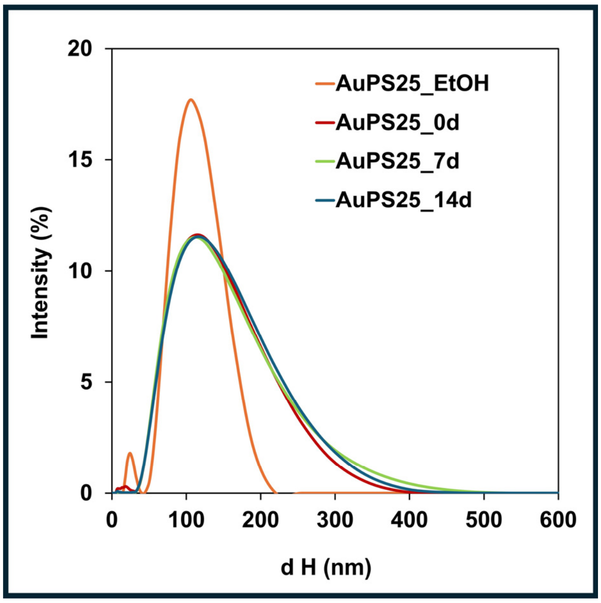

Supplementary Figure S2. Intensity-based size distribution of un-aged and aged AuPS25 NPs.

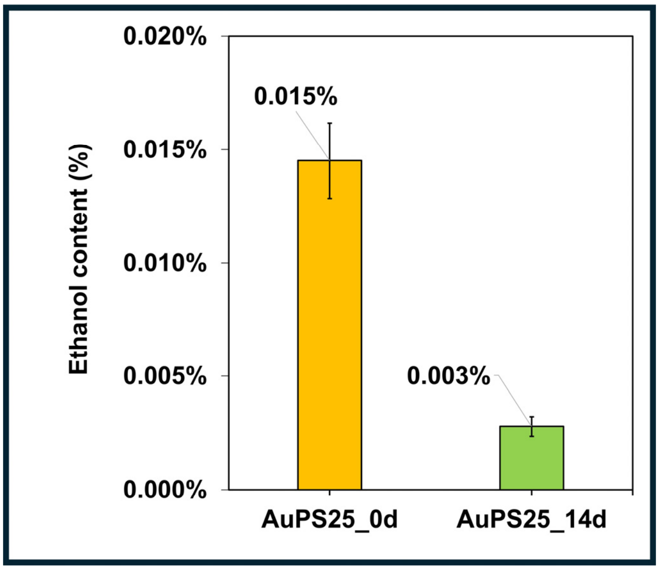

Supplementary Figure S3. Ethanol content assessment of un-aged and 14-day-aged AuPS25 NPs.

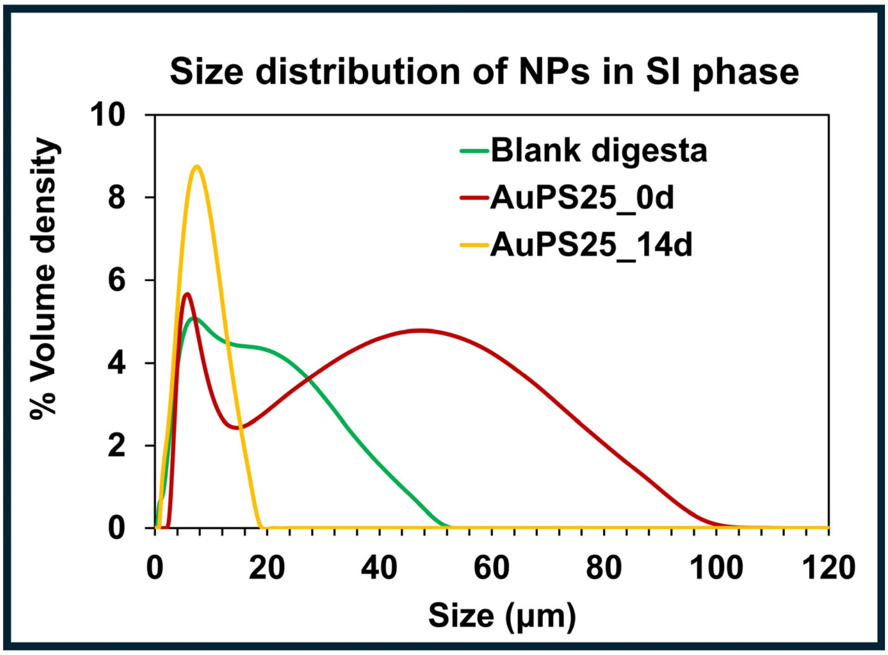

Supplementary Figure S4. Volume-weighted size distributions of final small intestinal digesta of water containing aged and un-aged AuPS25 NPs.

Reference

1. Park SC, Lim JY, Jeen YT, Keum B, Seo YS, Kim YS, et al. Ethanol-induced DNA damage and repair-related molecules in human intestinal epithelial Caco-2 cells. Mol Med Rep. 2012;5(4):1027-32; doi: 10.3892/mmr.2012.754.
2. Wang Y, Tong J, Chang B, Wang B, Zhang D, Wang B. Effects of alcohol on intestinal epithelial barrier permeability and expression of tight junction-associated proteins. Mol Med Rep. 2014;9(6):2352-6; doi: 10.3892/mmr.2014.2126.
3. Beltran-Huarac J, Zhang Z, Pyrgiotakis G, DeLoid G, Vaze N, Demokritou P. Development of reference metal and metal oxide engineered nanomaterials for nanotoxicology research using high throughput and precision flame spray synthesis approaches. NanoImpact. 2018;10:26-37; doi: 10.1016/j.impact.2017.11.007.
4. Kharaghani D, DeLoid GM, He P, Swenor B, Bui TH, Zuverza-Mena N, et al. Toxicity and absorption of polystyrene micro-nanoplastics in healthy and Crohn’s disease human duodenum-chip models. Journal of Hazardous Materials. 2025;490:137714; doi: 10.1016/j.jhazmat.2025.137714.
5. Li Y, Boraschi D. Endotoxin contamination: a key element in the interpretation of nanosafety studies. Nanomedicine. 2016;11(3):269-87.
6. Li Y, Fujita M, Boraschi D. Endotoxin contamination in nanomaterials leads to the misinterpretation of immunosafety results. Frontiers in immunology. 2017;8:472.
7. Guarino V, Perrone E, Zizzari A, Bianco M, Giancane G, Rella R, et al. Controlling endotoxin contamination in PDMS-based microfluidic systems for organ-on-chip technologies. Polymer Testing. 2025;147:108795; doi: 10.1016/j.polymertesting.2025.108795.
8. Hilgendorf C, Spahn-Langguth H, Regårdh CG, Lipka E, Amidon GL, Langguth P. Caco-2 versus caco-2/HT29-MTX co-cultured cell lines: permeabilities via diffusion, inside-and outside-directed carrier-mediated transport. Journal of pharmaceutical sciences. 2000;89(1):63-75; doi: 10.1002/(SICI)1520-6017(200001)89:1<63::AID-JPS7>3.0.CO;2-6
9. Artursson P, Palm K, Luthman K. Caco-2 monolayers in experimental and theoretical predictions of drug transport1PII of original article: S0169-409X(96)00415-2. The article was originally published in Advanced Drug Delivery Reviews 22 (1996) 67–84.1. Adv Drug Deliv Rev. 2001;46(1):27-43; doi: 10.1016/S0169-409X(00)00128-9.
10. Ensign LM, Cone R, Hanes J. Oral drug delivery with polymeric nanoparticles: the gastrointestinal mucus barriers. Adv Drug Deliv Rev. 2012;64(6):557-70; doi: 10.1016/j.addr.2011.12.009.
11. Mahler GJ, Shuler ML, Glahn RP. Characterization of Caco-2 and HT29-MTX cocultures in an in vitro digestion/cell culture model used to predict iron bioavailability. The Journal of nutritional biochemistry. 2009;20(7):494-502; doi: 10.1016/j.jnutbio.2008.05.006
12. Cabellos J, Delpivo C, Fernández-Rosas E, Vázquez-Campos S, Janer G. Contribution of M-cells and other experimental variables in the translocation of TiO2 nanoparticles across in vitro intestinal models. NanoImpact. 2017;5:51-60; doi: 10.1016/j.impact.2016.12.005.
13. Lai YH, D'Souza MJ. Microparticle transport in the human intestinal M cell model. Journal of drug targeting. 2008;16(1):36-42; doi: 10.1080/10611860701639848
14. Gullberg E, Leonard M, Karlsson J, Hopkins AM, Brayden D, Baird AW, Artursson P. Expression of specific markers and particle transport in a new human intestinal M-cell model. Biochemical and biophysical research communications. 2000;279(3):808-13; doi: 10.1006/bbrc.2000.4038
15. DeLoid GM, Wang Y, Kapronezai K, Lorente LR, Zhang R, Pyrgiotakis G, et al. An integrated methodology for assessing the impact of food matrix and gastrointestinal effects on the

biokinetics and cellular toxicity of ingested engineered nanomaterials. Particle and Fibre Toxicology. 2017;14(1):40; doi: 10.1186/s12989-017-0221-5.
16. Mahler GJ, Esch MB, Tako E, Southard TL, Archer SD, Glahn RP, Shuler ML. Oral exposure to polystyrene nanoparticles affects iron absorption. Nature nanotechnology. 2012;7(4):264-71; doi: 10.1038/nnano.2012.3.
17. Araújo F, Sarmento B. Towards the characterization of an in vitro triple co-culture intestine cell model for permeability studies. International journal of pharmaceutics. 2013;458(1):128-34; doi: 10.1016/j.ijpharm.2013.10.003.
18. Antunes F, Andrade F, Araújo F, Ferreira D, Sarmento B. Establishment of a triple co-culture in vitro cell models to study intestinal absorption of peptide drugs. European Journal of Pharmaceutics and Biopharmaceutics. 2013;83(3):427-35; doi: 10.1016/j.ejpb.2012.10.003.
19. Kharaghani D, DeLoid GM, Bui TH, Zuverza-Mena N, Tamez C, Musante C, et al. Ingested Polystyrene Micro-Nanoplastics Increase the Absorption of Co-Ingested Arsenic and Boscalid in an In Vitro Triculture Small Intestinal Epithelium Model. Microplastics. 2025. doi: 10.3390/microplastics4010004.
